# Beyond Just Bacteria: Functional Biomes in the Gut Ecosystem Including Virome, Mycobiome, Archaeome and Helminths

**DOI:** 10.3390/microorganisms8040483

**Published:** 2020-03-28

**Authors:** Ravichandra Vemuri, Esaki M. Shankar, Marcello Chieppa, Rajaraman Eri, Kylie Kavanagh

**Affiliations:** 1 Department of Pathology, Section of Comparative Medicine, Wake Forest School of Medicine, Medical Center Boulevard, Winston-Salem, NC 27157, USA; kkavanag@wakehealth.edu; 2 School of Basic and Applied Sciences, Central University of Tamil Nadu, Thiruvarur 610005, India; shankarem@cutn.ac.in; 3 National Institute of Gastroenterology “S. de Bellis”, Research Hospital, 70013 Castellana Grotte (Bari), Italy; transmed@irccsdebellis.it; 4 School of Health Sciences, College of Health and Medicine, University of Tasmania, Launceston 7248, Australia; rajaraman.eri@utas.edu.au

**Keywords:** gut microbiota, virome, fecal virome transplants, mycobiome, archaeome, helminths

## Abstract

Gut microbiota refers to a complex network of microbes, which exerts a marked influence on the host’s health. It is composed of bacteria, fungi, viruses, and helminths. Bacteria, or collectively, the bacteriome, comprises a significant proportion of the well-characterized microbiome. However, the other communities referred to as ‘dark matter’ of microbiomes such as viruses (virome), fungi (mycobiome), archaea (archaeome), and helminths have not been completely elucidated. Development of new and improved metagenomics methods has allowed the identification of complete genomes from the genetic material in the human gut, opening new perspectives on the understanding of the gut microbiome composition, their importance, and potential clinical applications. Here, we review the recent evidence on the viruses, fungi, archaea, and helminths found in the mammalian gut, detailing their interactions with the resident bacterial microbiota and the host, to explore the potential impact of the microbiome on host’s health. The role of fecal virome transplantations, pre-, pro-, and syn-biotic interventions in modulating the microbiome and their related concerns are also discussed.

## 1. Introduction

The human gut contains a collection of microbes that include commensal, symbiotic, and pathogenic bacteria, as well as fungi, viruses, archaea, and helminths. Collectively, the microbes in the gut are known as gut microbiota, and their respective genomes are collectively known as the gut microbiome [1]. The normal human gut is unique to each individual and influenced by factors such as diet, early-life microbiota exposure, changing hygiene status, pollution, socioeconomic status, and other environmental factors [2,3]. During the past decade, the gut microbiota has been implicated as an essential factor in the pathogenesis of inflammatory bowel disease (IBD), cancer, cardio-metabolic diseases, obesity, and diabetes [2,3,4,5,6].

**Figure 1:**
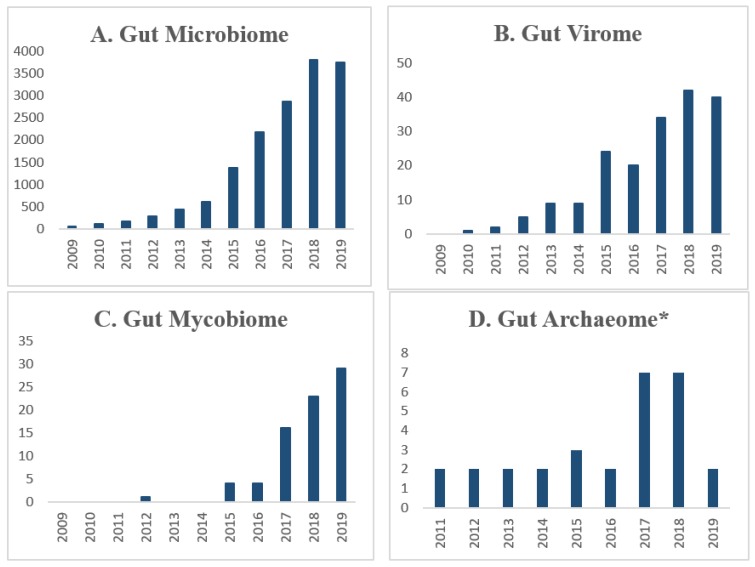
Scientific publications on various microbial communities of the gut microbiome. The number of peer-reviewed scientific publications for (**A**) microbiome, (**B**) virome (**C**) mycobiome and (**D**) archaeome studies. The list of peer-reviewed literature was collated in a non-systematic manner from the web of science (WOS) all database collection (MEDLINE, Inspec, Biological abstracts, scIELO, KCI, WOS core collection, Russian science index) from 2009 to 2019. The research of literature was performed using keywords like "microbiome”, “gut microbiome”, “gut virome”, “virome”, “gut fungi”, and “gut mycobiome”. * For careful selection of gut archaeome studies, we used PubMed only with keywords like “gut archaea”, “gut archaeon” and “gut archaeome/archaeon” and “archaeome” which includes whole microbiome analysis studies but not limited to only archaeome. Document types excluded were review of the literature (including systematic and meta-analysis), case studies, reports, and abstracts in conferences, workshops, and book chapters.

The human gut microbiome diversity is characterized in ecological terms by its species evenness (the number of different kinds of species present in an ecological community) and richness (the number of different species represented in an ecological community) [7]. Next-generation sequencing (NGS) revolutions enabling the 16S DNA-based quantitative identification of enteric bacteria sparked numerous microbiome-wide studies that correlated gut bacterial diversity and its composition shifts, termed as ‘dysbiosis’, with human disease [8]. Most of the available research literature on the microbiome has focused on bacteria or bacteriome but not the other components [4]. The number of studies on the gut microbiome (many correspond to bacteriome only) have skyrocketed, reaching up to 3500 papers in 2018 (Figure 1) (Supplemental Table S1). Although bacteria dominate microbial communities, viruses, archaea, and fungi may also play pivotal roles in maintaining the gut homeostasis [9], as evidenced by a more recent steep increase in studies assessing the role of viral and fungal components of the microbiome. However, the viral and fungal biomes remain relatively uncharacterized thus far, due to their lower abundance as well as lack of optimized tools and curated reference databases for their identification and classification [10,11].

Owing to advances in NGS technologies, researchers are now starting to understand the dark matter of the microbiota as summarized in Table 1. Emerging studies indicate that the gut virome mainly includes DNA or RNA bacteriophages [12]. In addition, fungi, which primarily comprises *Aspergillus*, *Candida*, *Fusarium*, *Penicillium*, and *Saccharomyces* [13], and likewise gut archaea, especially methanogens [14], represent notable members of the microbiome. Data on the role of helminth species in human health is very limited and more population-based studies are required to evaluate their role in the gut [15,16]. Clearly, though, viruses, fungi, archaea, and helminths also comprise a significant composition of the gut ecosystem, and their existence could account for host–microbe interactions [12]. In this review, we used human and animal studies to discuss gut virome, mycobiome, archaeome, helminths, and their interaction and health impacts. 

## 2. Gut Virome 

The bacterial and fungal communities residing in the gastrointestinal (GI) tract have undeniable far-reaching effects in regulating host health [1,12]. In contrast, the composition and dynamics of the gut virome are largely unknown, mainly due to limitations in identification. This is likely because of the lack of availability of viral genomes in the NCBI databases due to diverse global viral populations and their size. However, the impediments to identify and classify the virome have been now overcome with advancements in NGS, allowing us to make more substantial analyses of viromes [25]. Bioinformatics tools, such as the Metavir (versions 1 and 2) [26], bioinformatics pipeline VIROME (Viral Informatics Resource for Metagenome Exploration) [27], VIROMEScan [28], PHACCS (Phage Communities from Contig Spectrum) [29], CLAssification of Mobile genetic Elements (ACLAME) [30], VirusSeeker [31], the Phage SEED, and OptItDBA (Optimized Iterative de Bruijn Graph Assembly) have greatly improved the breadth and accessibility of virome analysis by improving assembly and annotating viromes against multiple annotated sequence databases, which improves the analytic capabilities beyond the constraints of individual sequence databases [32,33].

### 2.1. Gut Phages

The gut virome includes diverse commensal and pathogenic viruses that have abilities to infect host cells as well as other microbes, both avenues are able to directly affect the host’s health (Table 2). With a high inter-individual variation, the gut virome is mostly dominated by bacteriophages [34]. It also includes prophages, eukaryotic viruses, and retroviruses (Table 3). Very recently, studies on metagenomic sequencing of fecal DNA have begun to consistently unravel the extremely complex composition of the gut virome [10,35]. The DNA and RNA viruses that collectively make up the gut virome are at least comparable in number to bacterial cells, but on the gut mucosal surfaces and within the mucous layers; they may outnumber bacterial cells by 20:1 [36,37]. Each gram of human gut content is estimated to contain at least 10^8^–10^9^ virus-like particles, the vast majority of which are cross-assembly phage (DNA) phages belonging to the family Podoviridae [38,39]. Several studies have characterized the gut phage communities in newborn children, demonstrating a high richness and diversity and low stability during the initial days of life [17,40]. This diversity diminishes over the first two years, contrary to what has been observed in bacterial populations, which shift from low to high diversity [41]. Gut phages are primarily classified as members of the double-stranded (ds) DNA virus families such as Myoviridae, Podoviridae, and Siphoviridae within the order Caudovirales, or of the single-stranded (ss) DNA Microviridae family [12]. Recognizing the high abundance of phages in the gut, a Bacteroidetes-infecting phage family was identified and its founding phage alone represented up to 22% of all reads in the human gut metagenome project [42]. Another study characterizing Microviridae from healthy human donors also clustered with the Bacteroides and Prevotella prophages 72, suggesting that Microviridae could be an important viral family in the human gut. These prophages are considered lytic phages, which can integrate into bacterial hosts in an environment that encourages a temperate (lysogenic) viral-host lifestyle [39,41,43].

### 2.2. Enteric Viruses 

While the number of eukaryotic viruses present in the gut is lesser than that of phages, they are detected in metagenomic studies by PCR-based fecal shedding analyses in healthy individuals [45,63]. For example, viruses from the Anelloviridae, Picobirnaviridae, Adenoviridae, and Astroviridae families and species such as *Bocavirus*, *Rotavirus*, *Enterovirus*, and *Sapovirus* were identified in the fecal DNA samples of healthy children [41,45]. As the presence of eukaryotic viruses in the gut is far less well characterized, some studies have shed light on the dynamics of these viruses, with at least 16 different DNA viral families and 10 RNA families having been detected in gut samples [64]. These observations demonstrate that intestinal colonization with eukaryotic viruses, some of which are known to have pathogenic potential, can be tolerated without apparent symptomatic disease.

### 2.3. Role of the Gut Virome in Gastrointestinal Health and Disease

When considering the role of the enteric virome in the pathophysiology of GI diseases, the bridge from association to causation needs to be crossed. The gut virome, including enteric eukaryotic viruses and bacteriophages, can elicit chronic inflammation by infecting and killing the host cell as well as bacteria [65]. The first gut virome studies linked the presence or absence of certain viral components to diseases such as IBD and type 1 diabetes (T1D) [9,66]. The decreasing richness of gut bacteria occurring with IBD (both Crohn’s disease (CD) and ulcerative colitis (UC)) patients is well established [2]. In contrast, the fecal virome component’s richness was increased in those disease states when compared to healthy controls [67,68]. Specifically, the number of Caudovirales bacteriophages were increased and associated with viral richness relative to healthy controls [66,68]. Similar results were reported by another study that found reduced Caudovirales phages in healthy individuals when compared to CD and UC patients [51]. Studies of viral communities of diarrheic specimens for unknown causes in individuals from the Americas and Europe resulted in finding a common group of viruses that included *Anelloviruses*, *Picobirnaviruses,* and *Aichivirus* [69,70]. The role of these viruses, as well as if their presence is the cause or a consequence of a diseased GI tract, remains undetermined. In contrast to the richer gut virome observed in IBD patients, the gut viromes of individuals developing T1D were found to be less diverse than the ones from healthy controls, with the latter harboring significantly more viruses of the Circoviridae family [67], suggesting that viruses in the GI tract, such as Circoviridae in T1D, may alternatively exert beneficial functions for preserving host health [71].

### 2.4. Viromes in Fecal Microbial Transplants and Fecal Virome Transplants

Fecal microbiota transplantation (FMT) has played a significant role in treating recurrent *Clostridium* associated diarrhea. FMT or a nature-tailored probiotic treatment appears to restore normal or donor-like gut microbial (specifically bacterial) diversity. However, understanding the complex nature of gut microbiome, investigation into the presence and contribution of viromes in such fecal transplants has also begun, shown in Table 4. It is now certain that fecal microbiota contains a high abundance of viruses, primarily bacteriophages (90%) helping to repopulate gut bacterial diversity. As discussed, phages outnumber bacteria on mucosal surface and concerns persist about the unwanted transfer of pathogenic viruses from donor to recipient. The findings from a study by Chehoud and colleagues on FMT treatment in children with UC confirmed the transfer of viral-like particles [72]. Specifically, the members of Siphoviridae (temperate phages) were transferred with greater efficiency; however, none of the viral groups infected human cells. Zuo et al. investigated the presence and role of virome in patients with *C. difficile* infection (CDI) [73]. This particular study [73] confirmed the presence of phages from Caudovirales (tailed bacteriophages) before FMT treatment, but lower diversity, richness, and evenness compared to healthy controls. More recently, it was found that the impact of a successful FMT on the virome lasted for 12 months and resultant colonization of specific phages depended on donor-recipient combination [74]. This is consistent with a pilot study in which FMT devoid of bacteria was effective in CDI treatment, and increases the possibility that phages may be involved in the success of FMT [75]. 

In line with the studies mentioned above [75], a few researchers have recently developed a novel therapeutic intervention known as fecal virome transplantation (FVT), where only the viral component from FMT is transplanted [76,77,78]. Draper et al. confirmed the role of the virome in health and disease and showed that FVT, primarily consisting of phages, ameliorated antibiotic-induced bacterial dysbiosis [74]. FVT in mice resulted in a significant impact on not only abundance and diversity of bacteriome, but also the virome. The transplanted phages were able to colonize the gut and reshape the bacteriome similarly to a pre-antibiotic state. Likewise, a study by Rasmussen et al. utilized FVT in a type-2-diabetes and murine-obesity model [78], whereby only the virome in transplants from lean mice fed with a low-fat diet (LFD) to both obese mice (diet-induced obesity (DIO)) and mice fed a high-fat diet (HFD) + antibiotics, unlike Turnbaugh et al. [79]. FVT from lean donors partially reshaped the gut microbial composition in both of the recipient groups and decreased weight gain in DIO mice. In both these FVT studies [78], the gut viral composition was dominated by order Caudovirales and family Microviridae viruses. In another study, treatment with FVT (lytic and temperate gut phages) modulated gut microbial composition; the lytic phages enhanced the beneficial species of gut microbiota, and temperate phages stimulated the growth of commensal in the gut [76]. Interestingly, Hsu et al. confirmed that phages not only modulate the microbiome but also its associated metabolome [80]. In this particular study [80], gnotobiotic mice were subjected to lytic phages (FVT) after they were colonized with commensals, resulting in the finding that phage-led microbiome modulation was indeed due to intense microbe–microbe (intra- and inter-microbial) interactions which led to changes in the metabolome. More recently, Lin et al. compared FVT against FMT treatment on the ileal microbiome in mice fed with HFD [77], which promoted small intestinal bacterial overgrowth. They found reduced bacterial diversity in HFD mice receiving either FMT or FVT, compared to controls. Moreover, fecal transplants to control mice from HFD donor mice subjected to FVT shifted the ileal microbial composition similar to HFD mice, suggesting a causative role for FVT. Indeed, virome transplants could be beneficial as a therapy for many metabolic diseases related to gut microbiota, in addition to recurrent CDI. 

## 3. Mycobiome

Mycobiome, mycome, fungeome, or mycobiota is the collection of the fungal community and their respective genomes associated with the human body. Fungi are detected within the gut of many mammals, including humans, mice, rats, pigs, and numerous ruminant and non-ruminant herbivores [82]. NGS of the internal-transcribed-spacer (ITS) regions to identify the fungal ribosomal genes indicated the mycobiome as a third important dimension of the gut microbiomes [20]. However, the characterization of the mycobiome is complicated by the lack of comprehensive, accurate, and high-resolution taxonomic annotation within fungal databases [83]. Existing databases containing fungal targets include UNITE fungal ITS (1 and 2) database [84], Findley, ITSoneDB, RefSeq targeted loci (RTL), targeted host-associated fungi (THF) database, International Society for Human and Animal Mycology (ISHAM) ITS database, and SILVA [83,84,85,86,87,88,89,90,91]. The bioinformatics tools used in data analyses include MaAsLin [92] and MEGA software [93]. 

Several studies in recent years have detailed the importance of fungi within the human gut (Table 5) [82]. Shotgun metagenomics sequencing approaches suggest that fungi account for approximately 0.1% of the gut microbiome. An early study by Quin and colleagues that included 96 stool samples from healthy volunteers found 66 genera [94]. Fungi are detectable in all sections of the GI tract of about 70% of healthy adults, normally at up to 103 cfu per mL or g of intestinal contents [82]. Fungal genera commonly detected in mycobiome include *Candida*, *Saccharomyces*, *Fusarium*, *Debaromyces*, *Penicillium*, *Galactomyces*, *Pichia*, *Cladosporium*, *Malassezia*, *Aspergillus*, *Cryptococcus*, *Trichosporon*, and *Cyberlindnera* [95]. The potential roles played by these microbes in the human gut, however, is poorly understood.

Recent findings support the notion that a competitive association exists between bacterial and fungal microorganisms in the gut [6]. As an example, studies have shown that prolonged antibiotic usage is linked to fungal infection and overgrowth, particularly in the gut, and that germ-free mice are susceptible to infection with fungi such as *Candida* spp. [96]. Mycobiome dysbiosis is relevant in diseases such as IBD; the gut mycobiome of IBD patients has been characterized by reduced fungal diversity and a dysbiosis in community populations relative to healthy controls [97]. At the phylum level, the fungal ratio of Basidiomycota to Ascomycota was altered, with significantly higher relative abundance of Basidiomycota and a corresponding lower abundance of Ascomycota. More specifically, these trends have been attributed to higher relative abundances of the taxa *Candida*, Filobasidiaceae, and Malasseziales and a concurrent lower abundances of *Saccharomyces*, *Penicillium*, and *Kluyveromyces* [82,95]. 

Some emerging factors thought to be associated with composition of the mycobiota include host genotype and host physiology, including sex, age, and presence of comorbid conditions, lifestyles such as diet, hygiene, and occupation, and the immune system [98]. Diet represents a significant factor influencing the fungal mycobiome composition [6,99]. For example, *Candida* abundance is found to be strongly associated with the recent ingestion of carbohydrates [100]. However, further research is needed to establish the causality and explore the importance of additional factors that may influence the mycobiota, such as other lifestyle factors (e.g., exercise), medications (e.g., antibiotics or antifungals), and comorbid conditions.

## 4. Gut Archaea

Like bacteria, archaea are prokaryotes and categorized under the single-cell domain. Although morphologically they resemble bacteria, archaea have genes and metabolic pathways like other eukaryotes. The archaeal part of the gut microbiome is referred to as archaeome (Table 6). A range of human fecal NGS studies worldwide has reported archaeal prevalence up to 0.8 to 0.10% of the whole gut microbiome [5,94,110]. The most prominent archaeal microbes are methanogens (methane producers) and less prominent are halophilic (salt-loving microbes) archaea [110]. Molecular studies have indicated the presence of members of the orders Methanosarcinales, Thermoplasmatales, Methanomicrobiales, and Nitrososphaerales in the human gut microbiota; however, to date these microbes have not been isolated [14,111]. 

Methanogens are strict anaerobes that belong to the order Methanobacteriales, the most common genera being the closely related *Methaonbrevibacter* and *Methanosphaera* [110,112]. The abundance of methanogens increases from 0.03% to 11% of total gut microbes present between the proximal and distal colon [112]. *M. smithii* produces methane from the byproducts of bacterial fermentation and is present in up to 95% of fecal samples from human subjects [113]. Interestingly, *M. smithii’s* abundance was found to be stable over time, even after major changes in diet [14,114]. In the human gut, methanogenic archaea may impact host metabolism, especially energy homeostasis and may contribute to obesity [79], by syntrophic interactions with gut bacteria, which increase short-chain fatty acid production and contribute to an excess of calories available to the host [115]. Moreover, methanogens produce methane, shown to slow intestinal transit and lead to constipation [116]. In the case of IBD, methanogens like *M. smithii* and *Methanosphaera stadtmanae* have been most prevalent and shown to induce strong pro-inflammatory responses via monocyte-derived dendritic cells [117,118,119]. Halophilic archaea, or halophiles, are salt-loving microbes; the human gut environment is moderately salty, and a few IBD studies have isolated halophilic archaea from intestinal mucosal samples [120]. Currently, the studies on archaeal association with human disease are limited and further investigation is needed to elucidate its role in human health.

## 5. Helminths

Helminths are a type of multicellular parasitic intestinal worms found in various locations such as intestinal lumen, blood, or muscles of the host, and are usually referred to as macrobiome, and are known to be present in one-third of the global human population. Enteric helminths should be considered as a part of the gut microbiota as they co-reside in the gut with bacteria, viruses, and fungi [131]. Most of the helminth’s lifecycle is completed in the host’s intestine by disrupting the intestinal microbial ecosystem and imparting epithelial damage (Table 7). To control this damage, hosts promote the rapid expansion of intestinal epithelial cell numbers, mucus production, promotion of Th2 responses, and activation of T regulatory (Treg) cells to limit inflammation and increase wound healing capacity. Although most of these worms are considered parasitic, Allen et al. [132] suggested a symbiotic relation between helminths and host, whereby helminths are tolerated, and intestinal tissue damage is minimized. In a Malaysian population-based study, individuals with helminths had higher microbial diversity as compared to individuals without helminths [16,133]. Increasing evidence suggests that helminths also regulate mucosal inflammation [134,135]. Infection with helminths may lead to anti-inflammatory effects in the gut; specifically, in the case of IBD with *Trichiuris suis*, helminthic worms have ameliorated disease activities [136,137]. However, the exact mechanism of how helminths may protect the host from the development of IBD remains unknown. More studies on helminths and their relationship with host immune responses could lead to highly effective therapeutic strategies for human IBD, and other autoimmune disorders.

## 6. Cross-Kingdom Interactions

**Figure 2:**
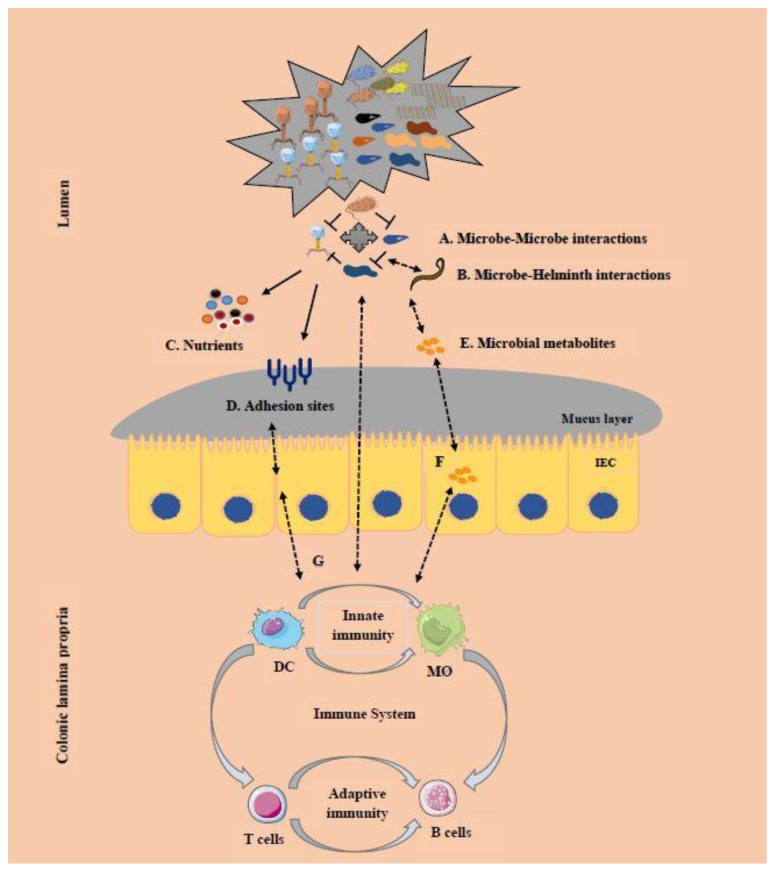
The microbial cross-kingdom interactions between members of microbiome. All the microbes in the gut interact with each other; Microbes interact with each other (**A**), with helminths (**B**) and their hosts, establishing trophic relationship (either symbiotic or parasitic). These interactions are categorized as competition or cooperation for survival, nutrients (**C**) and adhesion sites (**D**) on the mucosa. Most of these microbes produce specific metabolites (Short chain fatty acids) (**E**) and supply energy to intestinal epithelial cells (IEC), influencing the immune system (**F–G**), and overall homeostasis. DC = Dendritic cells; Mo = Macrophages.

Advances in ‘omics’ technologies have enabled researchers to better describe host–microbiome and microbiome–microbiome interactions, including the insight that different strains and species of microbes typically compete with each other for limited space and nutrients [145]. Emerging research suggests that the gut microbial system is densely colonized, promoting intense niche competitions for adhesion sites and nutrients (their limitations), and cooperation within and between microbial species [146]. Microbial competition occurs during initial encounters and gradually numbers reduce by co-exclusion, or niche separation or through spatial separation, enabling the coexistence of diverse communities [145]. Like environmental ecosystems, the gut microbial community is dynamic and regulated by cross-kingdom interactions (Figure 2). These intricate microbe–microbe and microbe–host–microbe interactions enable one microbial species to influence another and induce host response. Determining the role of the host in regulating these interactions and maintenance of homeostasis is vital to understanding species-level interactions in the gut. 

It is clear that interdependence between bacteriome, virome, and mycobiome exist and any imbalance in the gut microbial composition can impact overall human health [1,136]. For example, the phage can lyse commensal or pathogenic bacteria and drive bacterial evolution in the gut, molding the gut bacteriome [36]. In turn, the bacteriome inhibits pathogen colonization via competition, produces secondary metabolites, energizes host intestinal epithelium and enhances immune responses by host–microbe cross talk. For example, metabolites produced by bacteria can inhibit *C. albicans* colonization and translocation across the intestinal barrier [20]. Moreover, the efficacy of FMT was reduced due to the presence of *C. albicans* in donor stool, suggesting a strong relationship between fungal dysbiosis and FMT outcome [147].

Most of the studies on the microbiome suggest common themes between bacteriome, virome, mycobiome, and host genetics; however, there is no study indicating their direct relationships. Multiple factors determine the effect of the microbiome on homeostasis. Until recently, most of the studies have characterized interactions between bacteria and the host, while the relationships involving virome and mycobiome have received less attention. A few studies reported that interactions between components of microbiome and host could modulate the infectivity of viruses, such as the interaction between bacteria, poliovirus, and reovirus [10]. Bacteria can enable viral replication and cellular binding, facilitating chronic infections [37], or can also negatively affect viral infections. Most studies utilizing lactic acid probiotic bacteria such as *Lactobacillus* have efficiently blocked virus infections caused by rotavirus [148]. Conversely, the introduction of fungi can alter the composition of local, downstream, and upstream bacterial microbiota, while bacterial microbiota and the intestinal epithelium influence the ability of fungi to colonize the gut [13]. The commensal mycobiome-associated metabolites and products can also influence immune homeostasis, while helminths have immuno-regulatory effects evolved from host–parasite interactions [96]. Together, the above findings suggest that each member of the microbiome can individually affect the host immune system via basal stimulation (Table 8). 

**Figure 3:**
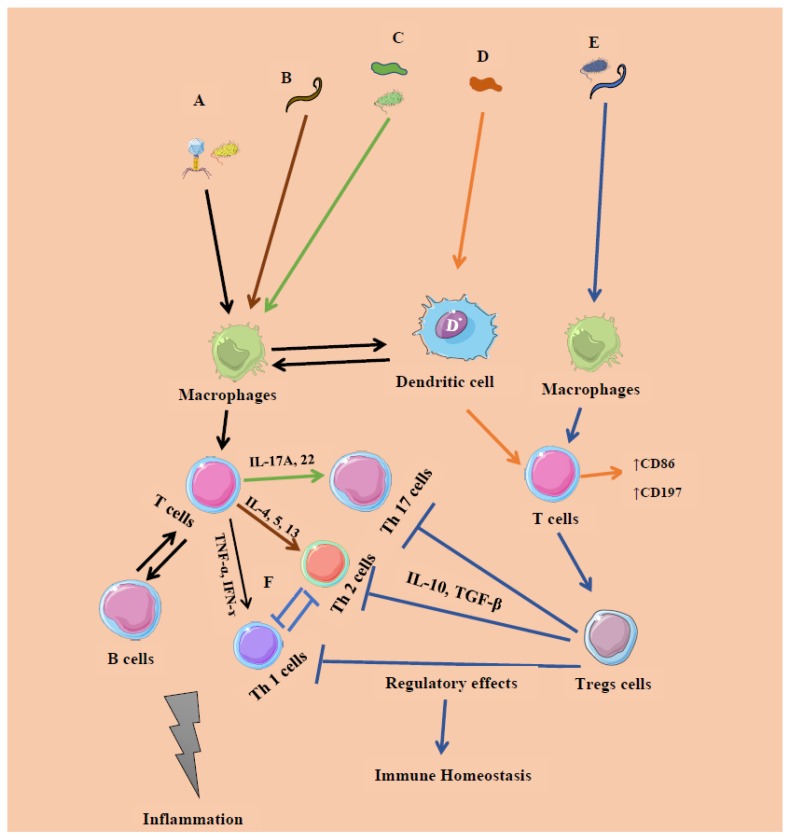
The known microbe–immune interactions in inflammation and homeostasis. **A**. Certain members of bacteria (intracellular, systemic commensals, Proteobacteria, and opportunist pathogens) and viruses induce inflammatory responses (IR 1) by promoting T cells and their subsets T helper 1 cells (Th 1) releasing pro-inflammatory cytokines such as interferon-gamma (IFN-γ), tumor necrosis factor-alpha (TNF-α), etc., initiated by microbe-associated molecular patterns (**Black arrow**). **B–C**. Segmented filamentous bacteria (extracellular), member of Fungi, and helminths (GATA 3) induce inflammatory responses initiated at the mucosal sites, which promote the expansion of T cells expressing Th 17 and Th 2 cells releasing Interleukins (IL) 17A, IL-22, and IFN-γ, respectively (**Brown and green**). **D**. Certain members of Archaea (*Methanomassiliicoccus luminyensis*, *Methanosphaera stadtmanae* and *M. smithii*) promote surface markers CD86, CD197 expressed on T cells releasing Monocyte-derived dendritic cells (MODC), type 1 IFN (**Orange**). All the factors involving **A–D** may lead to tissue damage and ultimately inflammation. **E–F**. Certain members of Clostridia, *Bacteroides fragilis*, archaea, and helminths induce regulatory responses by promoting Foxp3-expressing T regulatory (Tregs) cells, limiting the activation of Th1, Th2, and Th17 cells (**Blue**). This regulation and tolerance promote homeostasis.

Host immune responses are determined based on the characteristics of an individual member of the microbiome and its location (intra or extracellular) [149]. Bacteria, viruses, and fungi activate the innate part of the immune system via macrophages, which in turn actuate the adaptive immune system by employing T cells (pro-inflammatory) and their subsets such as T helper (Th) 1 and Th17 cells, respectively [150,151,152]. Similarly, helminths can activate macrophages and Th2 cells, leading to worm expulsion and inflammation. Excessive pro-inflammatory responses can cause damage to the host and thus are regulated by initiation tolerogenic regulatory responses via Treg cells [132] which reduce inflammation (Figure 3). Thus, the immunomodulatory effect of the microbiome promotes microbiome–immune tolerance and immune homeostasis. However, imbalance in the commensal microbial composition may play an important role in the development of autoimmune and chronic inflammatory responses. Trans-kingdom interplay, therefore, adds a layer of complexity in terms of host–microbial and immune homeostasis. Apart from bacteria, more definitively elucidating the roles of human gut virome, mycobiome, and helminths may have the potential to augment detection of disorders and act as disease markers. New developments in metagenomics, enrichment cultures, and bioinformatics tools are urgently required to improve our ability to define and characterize these biomes.

## 7. Modulation of the Microbiome and Related Concerns

To manipulate the microbiota, one should account for all the members of the gut microbial community; to that end, diet is considered a vital factor to positively modulate the gut microbiome. Different diets, such as LFD and HFD, have varied effects on each microbial community of the gut. For instance, *Bacteroides* and *Prevotella* genera were found to correlate with different diets inversely. Abundance of Bacteroides was associated with high fats, amino acids, and choline consumption, whereas *Prevotella* is correlated with carbohydrate consumption [153]. Additionally, the phage community of the gut is significantly altered by diet based upon pre-existing populations, indicating that individuals with the same diet may have similar, but not identical, viromes [50]. Similar trends were observed in both mycobiome and archaeome. Recent and high consumption of carbohydrates is directly linked to abundance of *Candida*, whereas a long term patterns of carbohydrates consumption is linked to profusion of *Methobrevibacter* spp. [100]. 

A growing body of evidence demonstrates the use of probiotics, prebiotics, synbiotics (pre- and probiotics together), FMT, and recently FVT, to modulate the microbiome [1,2,3]. Although these therapies produced promising outcomes in a few clinical and experimental models, most of them have not been consistent and may not provide consistent clinical benefits. For example, probiotics (single or multi-strained) are considered natural, safe, and beneficial modulators of the gut microbial composition, but their efficacy is strain- and dose-dependent and not all probiotics function against all disorders and diseases [154]. Fundamentally, to confer health benefits a probiotic needs to survive the acidic gastric digestion, other digestive enzymes, reach the intestine and colonize on the intestinal epithelium in good numbers [114]. In most of the probiotic trials, the vehicles for probiotic intake used are yoghurt, biscuits, bars, and capsules, per oral, which is not consistent across the studies and can potentially confound the outcomes [155]. While the exact probiotic mechanism is still unknown, the route of administration and dosage form may influence colonization. The colonization of probiotic strains is also dependent on competition or cooperation (microbe–microbe interactions) with the resident microbiota. Recently, several commercially available probiotics are increasingly multi-strain rather than a single strain [155], but how these multi-strains work and interact with each other to confer health benefits is still unknown. Specific probiotic strains and their combinations need to be further investigated to deploy them to their full potential. 

Synbiotics, combinations of pro- and prebiotics, are also arbitrarily chosen in many studies [156]. Sometimes the selected probiotic may not be able to even ferment the selected prebiotic strains [2,156]. The components of synbiotics are rarely tested individually in clinical or pre-clinical studies. Similarly, FMT is currently being explored for virome and mycobiome in the donor fecal samples and but also has not undergone rigorous component-based testing. 

## 8. Conclusions

It is imperative to understand that gut microbiome/macrobiome is not limited to bacteria but also viruses, fungi, and helminths. All these microbes interacting with each other, and with the host, in combination or alone, influence the health of the host. Advances in NGS technologies enable us to study the whole microbiome in an integrative way by exploring the taxonomic profiles and functional attributes of the various microbial communities. Considerable further study will be required to understand the development and regulation of gut microbial communities and the factors mediating the balance between long-term stability and dynamic response to the environment.

## Figures and Tables

**Table 1:** Summary of a few significant clinical studies on the viral, fungal, and archaeal microbiomes.

Microbial Component	Samples	Sorting/Analysis Method	Reads/Contigs */Sequences	Ref.
Viral	21	Hidden Markov Models/STAR	Assembled: 107,307 contigs. Total taxonomically assigned: 12,751 contigs (29.62% only)	[17]
	10		Assembled: 294,211 contigs Alignment of reads to contigs: 57,721 Final catalogue: 39,254 contigs	[18]
	32	CD-Hit-est	Total reads: 1,386,331 (32 datasets) Date normalized: 14,000 reads/dataset Annotated yield: 5004 contigs	[19]
Fungal	317 (147 subjects)	Internal Transcribed Spacer 2 (ITS)	Total reads per 1 sample/subject: 756,316 reads Total reads/sample: 17,189	[20]
	14	ITS1 (IonPGM, MiSeq, PacBio Sequence comparisons)	IonPGM: 219,756 reads MiSeq: 181,436 reads PacBio: 2984/sample Total: 41,776 reads	[21]
	49	ITS	106,185 reads	[22]
Archaeal (Methanogens)	21	16S gene analysis	1521 sequences	[23]
	10		10,000 reads/sample	[24]
	49		109,561 reads	[22]

* Contigs = either a DNA segment or set of overlapping DNA sequences.

**Table 2:** Summary of significant study findings on the gut virome.

Model Type	Study Type	Findings	Ref
**Human**			
Healthy infant	1 week old infant N = 1	Taxa identified: *Siphoviruses* and prophages (the majority (72%)	[41]
Infants/mothers (healthy)	Healthy adult female monozygotic co-twins and their mothers at three time points over a one-year period N = 12	Eukaryotic viral genomes: 73.3%, phages and prophages: 25.8% dsDNA phage (Caudovirales): 76.9%	[19]
	N = 8 (4 twin pairs)	Taxa identified: Siphoviridae, Inoviridae, Myoviridae and Podoviridae	[43]
	N = 24 longitudinal fecal samples	Taxa identified: Microviridae, Podoviridae, Myoviridae, and Siphoviridae	[44]
Healthy vs. malnourished infants/children	Time-series from fecal samples (Malawian) healthy control (HC)= twins, Mal Nutr = 12 twin pairs	↑ Anelloviridae (ssDNA eukaryotic viruses) in healthy infants and children (up to 15–18 mo), With age: ↑ Alpavirinae (ssDNA phages), ↑ Siphoviridae in 0 to 10 mo of age and then slowly decrease.	[45]
Obese children	N = 20 (HC = 10, Obese = 10)	Obese to HC: ↑ Human herpesvirus 4	[46]
Human (Healthy)	Longitudinal metagenomics analysis (Ireland) of fecal viruses N = 6 (3 Males, 3 Females)	Taxa identified: ↑ Virulent crAss-like and Microviridae bacteriophages	[18]
	Uncultured viral community from human feces. N = 1	Taxa identified: Bacteriophages A118 of *Listeria monocytogenes*, E125 of *Burkholderia thailandensis*, and bIL285 of *Lactococcus lactis*	[47]
	Analysis of the RNA viruses (N = 2)	Pepper mild mottle virus = 10^9^ virions/gram of dry fecal matter, ↑ RNA viruses	[48]
Virome of the ELDERMET	First-ever in elders (>65 yrs)	Taxa identified:Gokushovirinae (Microviridae)	[49]
Diet variations	sequencing (N = 6) high-fat/low-fiber diet	High fat to low fiber: Siphoviridae (18%), 686 (10%) to Myoviridae, 344 (4.8%) to Podoviridae, 68 (0.9%) to Microviridae, and 0.4% others	[50]
Human Virome (IBD)	Virus-like particle preparations on the rectal mucosa (N = 167, (UC = 91; HC = 76)). (Chinese study)	UC to HC = ↓ mucosal Caudovirales diversity, richness and evenness relative to HC	[51]
	metagenomics sequencing of stool filtrates using the Roche 454 platform (UK)	The viromes of CD and UC patients were disease- and cohort-specific. ↑ Caudovirales bacteriophages compared to HC	[52]
T2D vs. HC	Type II diabetes (T2D) patients (*n* = 71) and normal Chinese adults (HC) *n* = 74)).	T2D to HC: ↑ phages (Siphoviridae (55.3 ± 9.8%) Myoviridae (21.7 ± 9.9%), Podoviridae (10.6 ± 8.4%))	[53]
HIV study	(N = 122, untreated HIV = 42, HIV ART= 40, HIV uninfected= 40)	HIV to uninfected: ↑ Anelloviridae, *Adenoviruses*	[54]
**Animal**			
Gorilla simian immunodeficiency virus infection (SIV).	(N = 22, SIV = 11, HC = 11)	SIV to HC: ↑ Herpesviridae and Reoviridae Identified: Siphoviridae, Myoviridae and Podoviridae	[55]
Rodents	N = 314 wild rodent	Taxa identified: *Parvovirus, Dicistrovirus, Iflavirus,* and *Iridovirus*	[56]
	viral RNA and DNA in the feces of 105 wild rodents	Taxa identified:Circoviridae, Picobirnaviridae, Picornaviridae, Astroviridae, Parvoviridae, Papillomaviridae, Adenoviridae, and Coronaviridae.	[57]
	viral content in rat fecal matter (N = 29)	Picornaviridae	[58]
Mouse	N = 416 mice	Taxa identified: *Parvovirinae, Chapparvovirus, Polyomavirus, Astroviruses, Sapovirus, Picornavirus*	[59]
Mouse model of IBD	C57BL6/J mice (HC) and C57BL6/J *Rag1^-/-^* mice (IBD induced)	IBD to HC= ↑ Spounaviridae, ↓ Clostridiales phages	[60]
Gulf War illness (GWI) mouse model (IBD)	(N = 22, HC = 11, GWI = 11)	GWI mice to HC = ↓ Microviridae bacteriophages, ↑ Siphoviridae and Myoviridae bacteriophages	[61]
Gnotobiotic mouse model of phage-bacterial host dynamics	(N = 5 per group).	T7 phages are undetectable for 1 wk in germ-free animals before they rise in abundance after gavage of a bacterial host.	[62]

↑ increase; ↓ decrease; HC = healthy control; IBD = inflammatory bowel disease; ss = single stranded; T2D = type 2 diabetes.

**Table 3:** Viral communities identified in the mammalian gut.

Phages	ds DNA	ss DNA	ds RNA	ss RNA
Microviridae	Papillomaviridae	Circoviridae	Reoviridae	Retroviridae
Podoviridae	Polyomaviridae	Anelloviridae		Togaviridae
Siphoviridae	Poxviridae			Astroviridae
	Adenoviridae			Virgaviridae
	Iridoviridae			Caliciviridae
	Marseilleviridae			

ds = double stranded; ss = single stranded.

**Table 4:** Summary of studies and their findings involving in viromes from fecal microbial transplantations (FMT).

Model	Study Details	Findings	Ref.
Infant IBD	N = 4, Infant UC = 3, HC = 1 (22 to 30 FMT treatments	UC to HC: ↑ Siphoviridae	[72]
Adult IBD	N = 15, UC= 9, HC = 8	UC to HC: ↓ DNA phage, richness of donor viromes ≠ outcome of therapy Most abundant: Anelloviridae, Circoviridae, Picobirnaviridae and Virgaviridae	[81]
*Clostridium difficile* infection (CDI)	N = 44, CDI= 24, HC = 20	CDI to HC: ↑ Caudovirales (may play a role in FMT efficacy in CDI)	[73]
Recurrent CDI (rCDI) 1-year follow-up	rCDI = 14; donors (D) = 3	rCDI to D: ↑ Caudovirales, Anelloviridae ↓ Microviridae	[74]

↑ = increase; ↓ = decrease; N = total; HC = healthy control; IBD = inflammatory bowel disease; UC = ulcerative colitis; CDI = *Clostridium difficile* infection; rCDI= recurrent CDI.

**Table 5:** Summary of significant studies and their findings on the gut mycobiome.

Model type	Study details	Findings	Ref
**Human**			
Mycobiome of Human microbiome project (HMP)	N = 317	Taxa identified: *Saccharomyces*, *Malassezia*, and *Candida*. (↓ Mycobiome diversity)	[20,101]
Mother/offspring (prospective cohort)	N = 298 pairs (mothers and offspring)	From Mothers to off springs: ↑ *Debaryomyces hansenii* (breast-feeding) ↑ *S. cerevisiae* (after weaning)	[101]
The New Zealand human healthy gut mycobiome	N = 21 healthy, non-obese (age: 18–65 yr)	Taxa identified: *Candida albicans*, *Candida parapsilosis*, and *S. cerevisiae*. **New species identified**: *C. bracarensis*, *Coniochaeta hoffmannii*, *Hanseniaspora* *pseudoguilliermondii*, *Aspergillus foetidus*, *A. tubingensis*, and *Paecilomyces* *dactylethromorphus*	[102]
Healthy Aging study: Gut mycobiome of elderly Danish people (Age: 65–81 yr)	N = 99 (age:65 to 81 yr)	**Phyla**: Ascomycota, Basidiomycota and Zygomycota **Genera**: *Penicillium, Candida, and Aspergillus*	[103]
Mycobiota among Eutrophic, overweight, and obese	N = 72, Eutrophic = 24, Overweight = 24, Obese = 24).	Eutrophic: Zygomycota and Basidiomycota, Overweight: Zygomycota and Basidiomycota, Obese: Zygomycota, Basidiomycota and *Syncephalastrum* sp. (Zygomycota) Taxa identified include: ↑ Ascomycota (species): *Paecilomyces* sp., *Penicillium* sp., *Candida* sp., *Aspergillus* sp., *Fonsecaea* sp., and *Geotrichum* sp. ↑ Basidiomycota (species): *Trichosporon* sp. and *Rhodotorula* sp. ↑ Zygomycota (species) *Rhizopus* sp. and *Mucor* sp.	[104]
Healthy Japanese gut Mycobiota	N = 14	Taxa identified: ↑ *Candida* and *Saccharomyces*	[21]
Intestinal mycobiome of patients with irritable bowel syndrome (IBS).	N = 57, HC = 18 healthy IBS = 39	*S. cerevisiae* and *C albicans* identified in all samples/groups	[105]
**Animal**			
Rat model of visceral hypersensitivity	N = 6	*S. cerevisiae* and *C. albicans* in all samples.	[105]
Bat	N = 14	↑ Ascomycota and Basidiomycota	[106]
Dog	N = 19, HC= 12, acute diarrhea (AD)= 7	Ascomycota (HC: 97.9% and AD: 98.2%) and Basidiomycota (HC: 1.0%, AD: 0.5%)	[107]
Mouse	Pancreatic ductal adenocarcinoma (PDA) and C57BL/6 mice (HC).	PDA to HC: ↑ *Malassezia* species promote PDA	[108]
Tibetan macaque	n.a	Taxa identified:Zygomycota, Chytridiomycota, Glomeromycota and Rozellomycota	[109]

↑ = increase; ↓ = decrease; N = total; HC = healthy control.

**Table 6:** Significant studies and their findings on the gut archaeome.

Model type	Study details	Findings	Ref
**Human**			
Healthy infants	N = 15, Cesarean section-delivered (CSD) = 8, Vaginally-derived(VD) = 7	CSD to VD: ↑ *Methanosphaera* spp.	[121]
Infants/mothers (healthy)	N = 8 (4 twin pairs)	Lipothrixviridae	[43]
Obese children	N = 20 (HC = 10, Obese = 10)	Obese to HC: ↑ *Methanobrevibacter* spp.	[46]
	N = 476	Obese to HC: ↑ *M. smithii*	[122]
Healthy adults	N = 8	*M. smithii* (99–100%)	[123]
	N = 15 (Finnish)	Taxa identified: *Methanobrevibacter*–specific	[124]
Population-based	Belgian Flemish Gut Flora Project (FGFP; discovery cohort; N = 1106) and the Dutch LifeLines-DEEP study (LLDeep; replication; N = 1135)	↑ *Methanobrevibacter* spp. in all samples	[125]
Aging study	N = 500	Taxa identified: *M. smithii* *Methanosphaera stadtmanae* *Methanomassiliicoccus luminyensis*	[126]
Archaea of ELDERMET study	N = 371	Taxa identified: Methanomassiliicoccales	[127]
Type 2 diabetes	N = 49 HC= 19, New (type 2) = 14, Known (type 2)= 16	NGTs to Known = ↓ *Methanobrevibacter*	[22]
Human IBD	N = 58, HC = 29, IBD = 29	IBD to HC: ↑ *M. stadtmanae*	[128]
	N = 108, HC= 47, IBD = 61	IBD to HC: ↓ *M. smithii*	[129]
**Animal**			
Human/ape study	Humans (N = 10) Apes: *Pan troglodytes* (chimpanzee = 14), *Pan paniscus* (bonobo = 18), *Gorilla gorilla* (gorilla = 20), and *Pongo pygmaeus* (orangutan = 8)	Taxa identified: *M. smithii* and *M. stadtmanae* in all samples (more in humans)	[24]
Rabbit cecal archaea	N = 40	Taxa identified: *Methanobrevibacter* and *Methanosphaera* spp. in all samples	[130]

↑ = increase; ↓ = decrease; N= total; HC = healthy control; IBD = inflammatory bowel disease; NGT= long-standing diabetic subjects.

**Table 7:** Summary of significant study findings on the role of helminths in health and disease.

Model Type	Study Details	Findings	Ref.
Rat model (2, 4, 6 Trinitrobenzene sulphonic acid (TNBS) induced colitis model	N = 24: HC = 6, *Schistosoma mansoni* group = 6, TNBS group = 6 and *S. mansoni* + TNBS group= 6	*S. mansoni* group: ↑ IL-2, IL-4 TNBS group: ↑ IL-2 ↑ T helper 1 (Th1) *S. mansoni* plus TNBS group: ↑ Th2 ↓ Th 1 (↓ Inflammation) Concurrent infection with *S. mansoni* significantly attenuates TNBS induced colitis in the rats.	[138]
Rhesus macaques with idiopathic chronic diarrhea (ICD) w/o *T suis* infection	N = 7, ICD = 5 ICD with *T suis* = 5, HC = 2	ICD group = ↓ Mucosal bacterial diversity, ↓ Th2 ICD with *T suis* = ↑ Mucosal bacterial diversity (Cyanobacteria), ↑ Th2	[139]
Human (Tetanus toxoid (TT) and *S mansoni* infection)	TT + *S. mansoni* = 11 HC= 5	TT + *S. mansoni* group: ↓ Interferon gamma (IFN-ɤ), ↑ Th 2 HC: ↑ IFN-ɤ ↓ Th1	[140]
Human (helminthic ova in the treatment of active IBD)	N = 4 (IBD), 2500 live *Trichuris suis* eggs/12 weeks/subject	IBD: ↑ Inflammation: ↑ Th1 *T. suis* treated: ↓ Inflammation: ↑ Th2 and ↓ Th1	[141]
Human (RCT *T suis* therapy for ulcerative colitis (UC))	N = 54, Therapy group= 30, HC = 24	Therapy group =↓ UC disease activity index (DAI)	[137]
Human (RCT *T suis* therapy for Crohn’s disease (CD))	N = 36, Treated group = 27, HC = 9	*T. suis* therapy was well tolerated	[142]
Human (*Necator americanus* therapy for CD)	N = 18, (inoculation)	CD: ↑ DAI *N. americanus* treated: ↓ CD (DAI)	[143]
Human (*T suis* therapy multiple sclerosis)	N = 5	Multiple sclerosis: ↑ Inflammation *T. suis* treated: ↑ IL-4, ↑ IL-10 (↓ Inflammation = ↑ Th 2?)	[144]

↑ = increase; ↓ = decrease; HC = healthy control; N = total, RCT = randomized control trial, TT = Tetanus toxoid, UC = ulcerative colitis, CD = Crohn’s disease, DAI = disease activity index, IL = interleukins; Th = T helper cells; IFN = interferon.

**Table 8:** The immune responses associated with different members of the microbiome ([1,149,150]).

Microbial Constituent	Response Type	Immune Cells/Expressions	Cytokines	Initiation
Bacteria (intracellular): Systemic commensals, Proteobacteria, pathogens	Inflammatory response (IR) 1	T helper 1 (Th 1) cells	Interferon gamma (IFN-ɤ), Tumor necrosis factor (TNF-α)	Microbe-associated molecular patterns (Pro-inflammatory)
Virus	IR 1	CD4, CD8 T cells	IFN-α/β, IFN-λ	
Segmented filamentous bacteria (extracellular), Fungi	IR 2	Th 17 cells	Interleukins (IL) 17A, IL-22	Mucosal epithelial cells
Helminths	IR 2	Th 2 cells	IFN-ɤ	GATA 3
Archaea ((*Methanomassiliicoccus luminyensis*, *Methanosphaera stadtmanae* and *M. smithii*))	Inflammatory/regulatory response	T cells- CD86, CD197	Monocyte-derived dendritic cells (MODC), type 1 IFN	Mucosal epithelial cells
Clostridia, *Bacteroides fragilis*, archaea and helminths	Regulatory response	T regulatory cells (Foxp3 + Tregs)	IL-10, Transforming growth factor beta (TGF-β)	Resolution of IR 1, 2

IR = Inflammatory response; Th = T helper cells; IFN = Interferon gamma; TNF = Tumor necrosis factor; IL = Interleukins; MODC = Monocyte-derived dendritic cells; TGF = Transforming growth factor.

## Supplementary Materials

The following are available online at https://www.mdpi.com/2076-2607/8/4/483/s1, Table S1: Number of studies published in Web of Science and PubMed databases related to gut microbiome, gut virome, gut mycobiome, gut archaeome and helminths.
